# Preparation and Characterization of Carbon Paste Electrode Bulk-Modified with Multiwalled Carbon Nanotubes and Its Application in a Sensitive Assay of Antihyperlipidemic Simvastatin in Biological Samples

**DOI:** 10.3390/molecules24122215

**Published:** 2019-06-13

**Authors:** Amir M. Ashrafi, Lukáš Richtera

**Affiliations:** Department of Microelectronics, Faculty of Electrical Engineering and Communication, Brno University of Technology, Technická 3058/10, CZ-616 00 Brno, Czech Republic; amirmansoor.ashrafi@mendelu.cz

**Keywords:** multiwalled carbon nanotube, carbon paste electrode, scanning electrochemical microscopy, simvastatin determination, mechanism study, biological sample

## Abstract

Determination of an antihyperlipidemic drug simvastatin (SIM) was carried out using a carbon paste electrode bulk-modified with multiwalled carbon nanotubes (MWCNT-CPE). Scanning electrochemical microscopy (SECM), scanning electron microscopy (SEM), and atomic force microscopy (AFM) were used for the characterization of the prepared electrodes. Different electrodes were prepared varying in mass percentage of MWCNTs to find out the optimum amount of MWCNTs in the paste. The MWCNT-CPE in which the mass percentage of MWCNTs was 25% (*w/w*) was found as the optimum. Then, the prepared electrode was used in a mechanistic study and sensitive assay of SIM in pharmaceutical dosage form and a spiked human plasma sample using differential pulse voltammetry (DPV). The prepared electrode shows better sensitivity compared to the bare carbon paste and glassy carbon electrode (GCE). The detection limit and the limit of quantification were calculated to be 2.4 × 10^−7^ and 8 × 10^−7^, respectively. The reproducibility of the electrode was confirmed by the low value of the relative standard deviation (RSD% = 4.8%) when eight measurements of the same sample were carried out. Determination of SIM in pharmaceutical dosage form was successfully performed with a bias of 0.3% and relative recovery rate of 99.7%. Furthermore, the human plasma as a more complicated matrix was spiked with a known concentration of SIM and the spiking recovery rate was determined with the developed method to be 99.5%.

## 1. Introduction

Simvastatin (SIM) is a long-established hydroxy-methylglutaryl coenzyme which was first introduced in 1998 [1]. It belongs to a group of drugs called statins. The oral administration of SIM is followed by its hydrolysis, generating the corresponding β-hydroxyacid form [2]. Then, the content of the low-density lipoprotein (LDL) cholesterol in plasma is reduced effectively by inhibition of 3-hydroxy-3-methylglutaryl-coenzyme A (HMG-CoA) reductase [3]. This enzyme acts by catalyzing the conversion of HMG-CoA to mevalonate, which is an early and rate-limiting step in the biosynthesis of cholesterol [4]. SIM is synthesized from a fermentation product of *Aspergillus terreus* [5]. In addition to the well-defined function of statins, some clinical trials showed that this group of drugs can protect against cardiovascular disease (CVD) through another mechanism [6]. Statins are among the most widely prescribed drugs in the world [7].

Due to the *in vivo* hydrolysis, the concentration of SIM in human plasma is very low (1–15 nM) [8,9,10,11]. Moreover, for the determination of SIM in plasma, a separation step is required. Thus, it is highly demanded to develop a sensitive analytical method for the determination of SIM in pharmaceutical dosage form and human plasma. The developed analytical methods for the determination of SIM consist of methods which provide the separation process, including high-performance liquid chromatography (HPLC) [2,12,13,14,15], gas chromatography (GC) [16], and capillary electrophoresis [17] in combination with detection methods including ultraviolet (UV) spectroscopy [12], fluorescence detection [14], mass spectrometry [2,13,15,16], and colorimetry [18].

However, the inherent features of electroanalytical methods, such as good selectivity and sensitivity, even in a complicated matrix, as well as being inexpensive, quick, and simple to execute, make them advantageous compared to the other analytical methods.

Several electroanalytical methods were applied for the determination of SIM, including adsorption of the SIM–Cd(II) complex at the mercury drop electrode and determination of SIM from the reduction peak of cadmium [19], cathodic square-wave stripping voltammetry using the adsorption of SIM onto the mercury electrode surface [20], direct voltammetry using the bare glassy carbon electrode (GCE) [21], GCE immobilized with sodium dodecyl sulfate [22], and GCE modified with a multiwalled carbon nanotube (MWCNT)–dihexadecyl hydrogen phosphate composite [23]. Although, these electroanalytical techniques showed good sensitivity, there are still some disadvantages, such as toxicity of the mercury and the short life of the surface-modified electrodes.

Blending the graphite powder with a pasting liquid forms a heterogeneous mixture which is known as carbon paste. Carbon paste is an attractive electrode material due to its electrochemical characteristics, including a very low background current, low ohmic resistance, cost-effectiveness, and simple electrode surface renewal [24]. Furthermore, the modification of the carbon paste can be simply carried out by adding the modifier to the paste [25,26,27]. To decrease the electrical resistance of the paste, room-temperature ionic liquids were used as a pasting liquid [28].

As a modifier, carbon nanotubes (CNTs) enhance the electrode performance by increasing the velocity of the electron transfer [29]. Moreover, reduced over-potential and improved selectivity are caused by modifying the electrode with CNTs [30]. In addition, CNTs possess a significant adsorption capability, which provides pre-concentration of the analyte at the electrode surface and consequently increases the sensitivity of the electrode. The sensitivity of the electrode is also improved due to the high surface area of CNTs [31]. 

This work aimed to prepare a carbon paste electrode (CPE) bulk-modified with MWCNTs for sensitive determination of SIM. Differential pulse voltammetry was used for the determination. The MWCNTs can increase the sensitivity of the concentration determination compared to the bare GCE and CPE. The preparation and surface renewing of the MWCNTs/CPE are rapid and easy. Moreover, compared to the previous studies, any toxic compounds such as mercury or other heavy metals are not used. Then, the developed electrode was used to determine SIM in human plasma and in the pharmaceutical dosage form. 

## 2. Results and Discussion

### 2.1. Scanning Electrochemical Microscopy (SECM) Analysis

**Figure 1:**
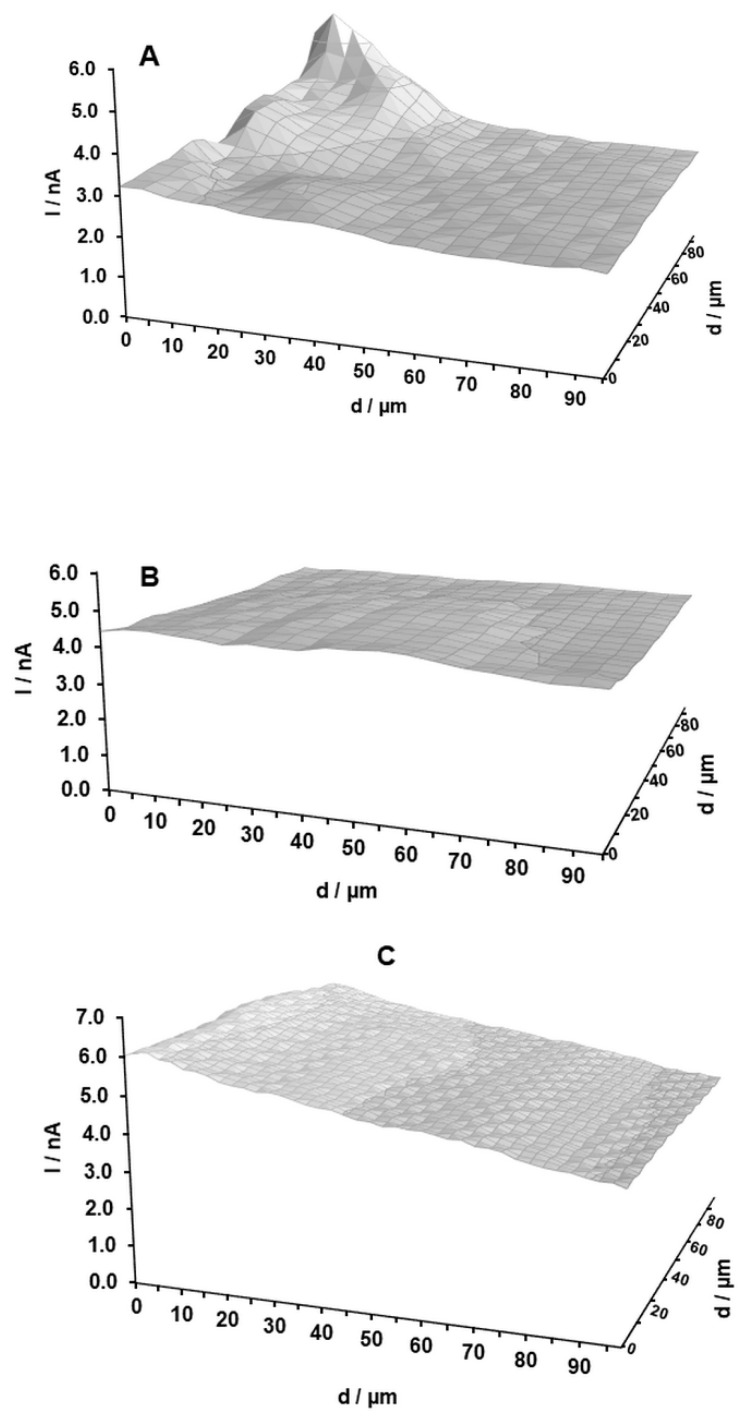
Scanning electrochemical microscopy (SECM) images of the multiwalled carbon nanotube carbon paste electrodes (MWCNT-CPEs): (**A**) 15% MWCNT-CPE; (**B**) 25% MWCNT-CPE; (**C**) 35% MWCNT-CPE. Measurement conditions: Pt ultra-microelectrode (UME) with 10 µm diameter and RG (the ratio of the insulator radius to the electrode radius) ≥ 10, FcOH 0.2 mM, and KCl 0.1 M; 0.6 V was applied at the tip; the scan rate of the tip to the substrate was 0.5 µm∙s^−1^. The tip approached to the substrate until $i_{T}$ = 1.25 $i_{T,\infty }$.

SECM images of three MWCNT-CPEs which differ in mass percentage of MWCNTs are shown in Figure 1. In SECM, a constant height and a homogeneous surface represent a slight current gradient, while the surfaces with lower homogeneity show a significant current gradient. The MWCNT-CPE with 25% MWCNTs shows a relatively homogeneous current which can be attributed to a higher degree of surface homogeneity. The current gradient shows some degrees of inhomogeneity in the cases of 15% and 35% MWCNTs, where some peaks or continuous changes in current profile can be observed. Recalling that the paste is a heterogeneous mixture of three components, it can be concluded that the MWCNT-CPE with 25% MWCNTs is optimal to achieve the highest homogeneity of the mixture. As a matter of experience, MWCNTs adsorb more oil compared to graphite. Hence, the 15% MWCNT-CPE is more lubricated. On the other hand, the 35% MWCNT-CPE paste is drier. Thus, the binding of the component is not efficient. The effect of the binder/graphite powder ratio on the carbon paste properties was investigated using different characterization methods. It was shown that, with a low amount of organic oil, the binding of the graphite powder is not well established, while a high amount of oil causes increased resistivity of the paste [32]. It is worth noting that each SECM analysis was repeated at least three times for each prepared electrode, and similar results were obtained.

### 2.2. SEM Analysis

**Figure 2:**
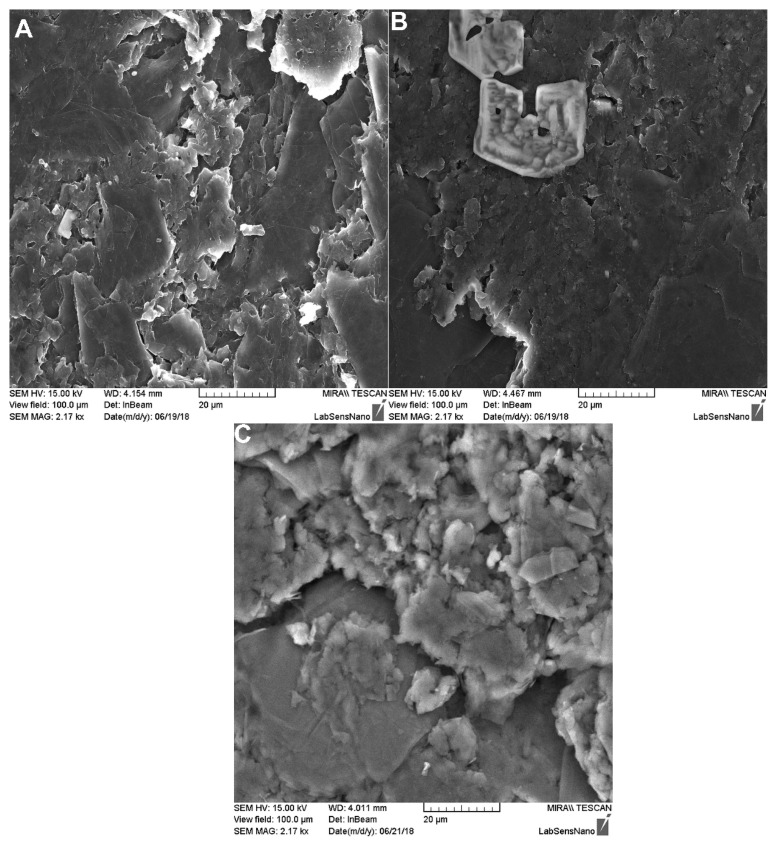
The SEM images of the prepared MWCNT-CPEs: (**A**) 15% MWCNT-CPE; (**B**) 25% MWCNT-CPE; (**C**) 35% MWCNT-CPE.

The SEM images of the three kinds of prepared electrodes are presented in Figure 2. The inhomogeneity of the 15% and 35% MWCNT electrodes can be attributed to more defects, which are visible on the electrode surface. The defects are formed due to the ineffective binding of the paste components because of the unjustified mass ratio of the components. The surface of the 25% MWCNT electrode shows less defects, which indicates higher homogeneity of the electrode surface.

Based on the obtained results, the 25% MWCNT-CPE was selected as the optimum electrode and used for the further experiments. It was used for the determination of SIM to get insight into its electroanalytical properties.

### 2.3. Atomic Force Microscopy (AFM) Analysis

**Figure 3:**
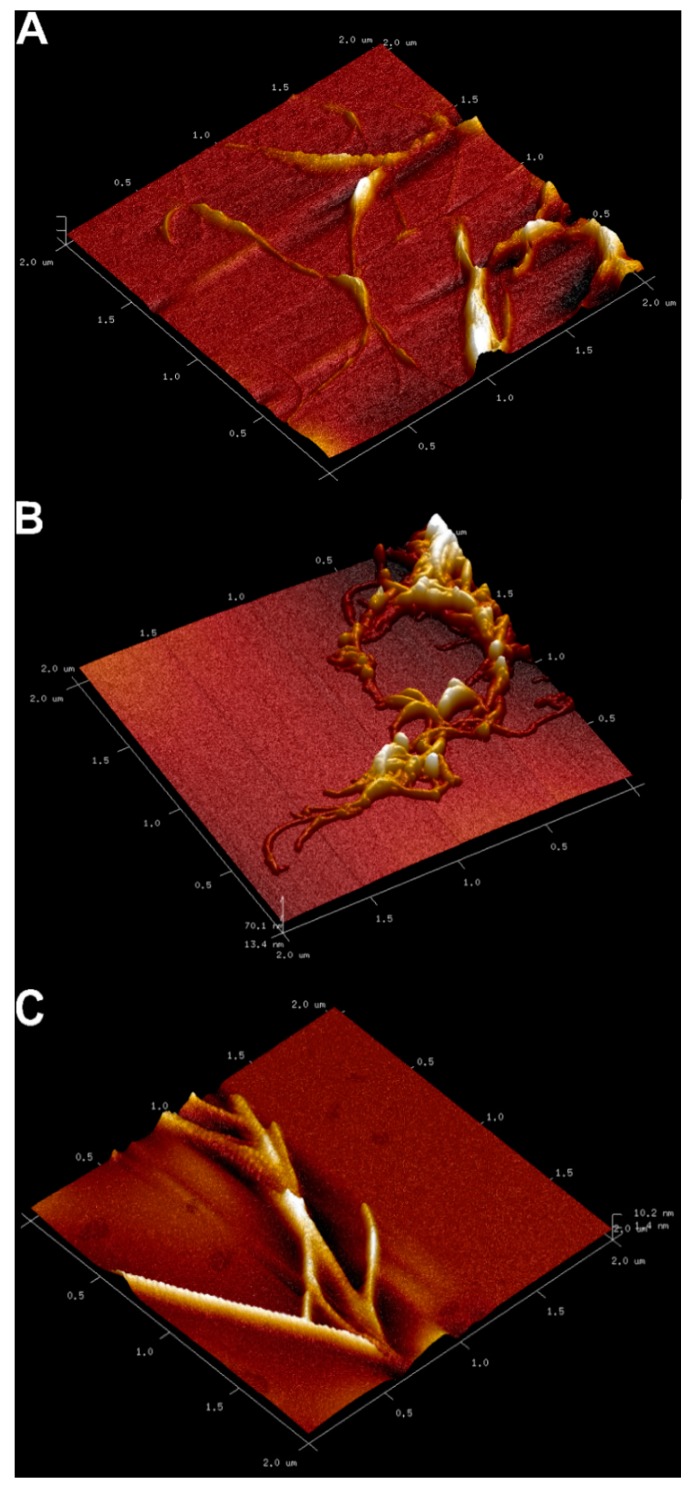
Atomic force microscopy (AFM) images of the prepared MW-CNTCPEs: (**A**) 15% MWCNT-CPE; (**B**) 25% MWCNT-CPE; (**C**) 35% MWCNT-CPE.

The three-dimensional (3D) AFM images of the developed electrodes are shown in Figure 3. The two-dimensional (2D) AFM images are also available in Figure S1. The presence of the MWCNTs on each prepared electrode is apparent. As seen, the MWCNTs are randomly distributed over the electrode surface. The bright spots around the MWCNTs are probably attributed to the mineral oil. As seen in Figure S1, the 35% MWCNT-CPE seems to be dry as the MWCNTs are not surrounded by the mineral oil. On the other hand, the MWCNTs in the 15% and 25% MWCNT-CPEs are well mixed with the mineral oil. The dryness of the electrode with 35% MWCNTs stems from the higher capacity of the MWCNTs in the adsorption of the mineral oil. Hence, the higher mass percentage of the MWCNTs requires more mineral oil.

### 2.4. Electrochemical Behavior of SIM on the MWCNT-CPEs

**Figure 4:**
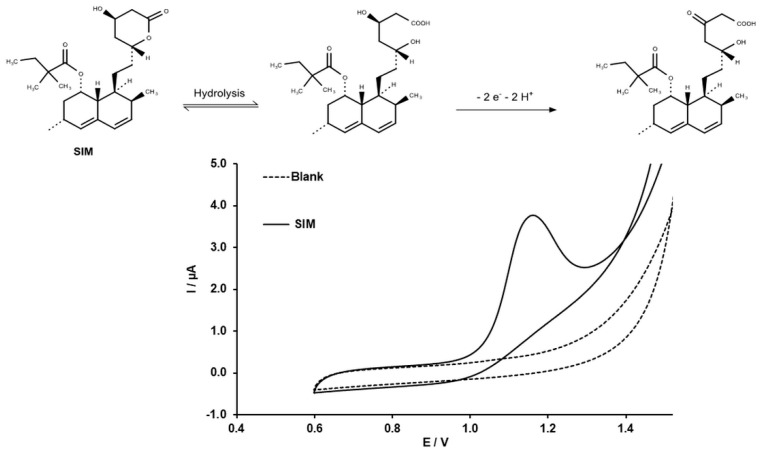
The CV of MWCNT-CPEs in 0.1 M H_2_SO_4_ at and the CV of 5 × 10^−4^ M simvastatin (SIM) in 0.1 M H_2_SO_4_ with MWCNTs, at a potential scan rate of 50 mV∙s^−1^. The inset shows the electrochemical oxidation mechanism of SIM.

The CV of SIM shows a distinctive irreversible oxidation taking place at 1.2 V in 0.1 M H_2_SO_4_ pH (Figure 4). The probable oxidation mechanism is also shown in Figure 4. To get more insight into the nature of the oxidation of the SIM, the pH dependence of the peak current and potential was studied.

**Figure 5:**
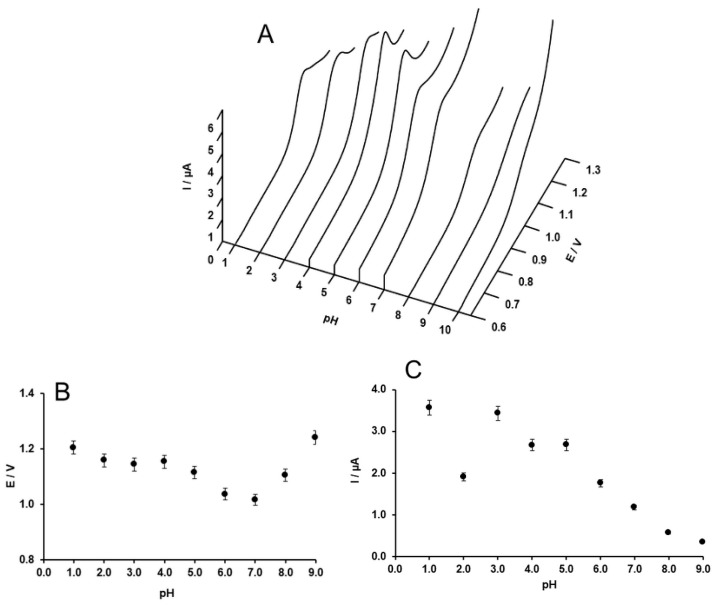
(**A**) Forward scan of CVs of 5 × 10^−4^ M SIM in 0.1 M H_2_SO_4_ at various pH values, with a potential scan rate 50 mV∙s^−1^; (**B**) pH dependence of the peak potential; (**C**) pH dependence of the peak current.

The changes in the peak potential and peak current are presented in Figure 5. As seen in Figure 5B, the peak potential shifts to less positive potentials upon increasing the pH from 1 to 7. In this pH range, the pH dependency of the peak potential follows Equation (1). (*R* = 0.948)

(1) $$E_{p}\;\left(\mathrm{mV}\right)=\;-30.693\cdot \mathrm{pH}+1240.5$$

However, in the pH range of 7 to 9, the peak potential increases with the pH. The slope of the pH dependency of the peak potential (pH = 1 to 7) is close to the theoretical value corresponding to the mechanism in which two protons and an electron are involved. However, the redox mechanism reported for the oxidation of SIM is a reaction involving two electrons and two protons. The abnormal variation of the peak potential with pH, which was also previously observed [21,23], might arise from the coexistence of SIM and its hydrolyzed product, since SIM might be hydrolyzed into simvastatin acid (β,δ-dihydroxyl acid). [23,33].

**Figure 6:**
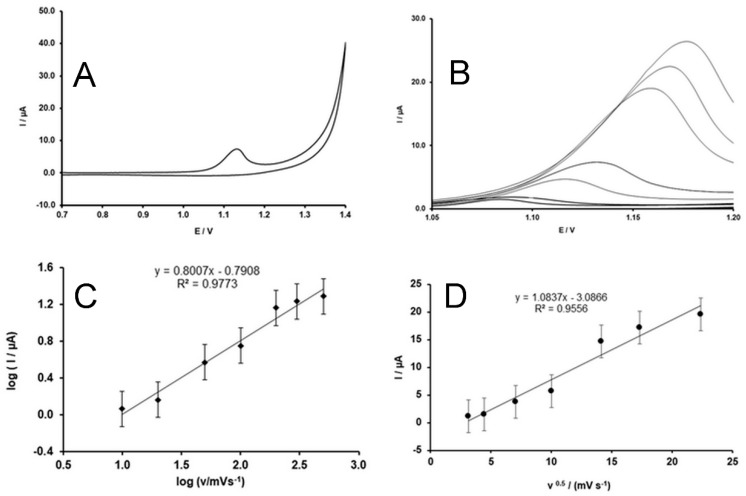
(**A**) CV of 5 × 10^−4^ M SIM in 0.1 M H_2_SO_4_ at 100 mV∙s^−1^; (**B**) the forward scan of CVs at various potential scan rates (10, 20, 50, 100, 200, 300, and 500 mV∙s^−1^) (only a potential range related to the peak is shown to see the peaks well); (**C**) scan rate dependence of the oxidation peak current in logarithmic coordinates; (**D**) dependence of the oxidation peak current on the square root of the scan rate.

The dependence of the oxidation peak current on the potential scan rate was also studied. The results are shown in Figure 6A,B. The oxidation peak shifts to a more positive potential upon increasing the scan rate, which confirms the irreversibility of the oxidation of SIM. The dependence of the peak current on the potential scan rate in logarithmic coordinates is shown in Figure 6C. The dependency follows Equation (2) (R=0.988).

(2) $$\log I\left(µA\right)=0.8007\cdot \log\nu \;\left({\mathrm{mVs}}^{-1}\right)-0.7908.$$

Furthermore, the dependency of the peak current on the square root of the scan rate (Figure 6D) is described by Equation (3) (R=0.977).

(3) $$I\left(µA\right)=1.0837\cdot \nu^{0.5}\;\left({\mathrm{mVs}}^{-1}\right)-3.0866.$$

Since the slope of Equation (2) is between the theoretical values of 0.5 and 1.0, which are the theoretical values of the diffusion- and adsorption-controlled mechanisms, respectively [34], it can be concluded that a combination of the two mechanisms is responsible for the mass transport to the electrode. However, since the slope is close to 1.0, the dominant mechanism is adsorption. Furthermore, the poor linearity of Equation (3) confirms that diffusion is not the main mechanism of the mass transport. 

The mechanism of the oxidation of SIM on the developed electrode was also investigated by performing 10 repetitive CVs of SIM in 0.1 M H_2_SO_4_ (Figure S2). The first cycle shows an oxidation peak at 1.10 V. Upon increasing the number of cycles, the peak current decreases and the peak potential shifts to more positive potentials. The shift of the oxidation peak to more positive potentials is due to the adsorption of SIM onto the electrode surface, which changes the physicochemical properties of the electrode surface, including its conductivity and catalytic capability.

The developed electrode was used to drive the Tafel plot in a 5 × 10^−4^ M solution of SIM at a potential scan rate of 50 mV∙s^−1^. The Tafel equation (Equation (4)) [35] was used to determine the transfer coefficient and the exchange current.

(4) $$\log\;I=logI_{0}\frac{\left(1-\alpha \right)nF}{2.30RT}\eta ,$$

where $I_{0}$ is the exchange current, I is the current, η is the overvoltage, and n is the number of transferred electrons. The transfer coefficient α was calculated to be 0.07 for this two-electron transfer process. The exchange current was also estimated from the intercept to be 1.8 × 10^−9^ A.

### 2.5. Analytical Parameters and Validation of the Method 

**Figure 7:**
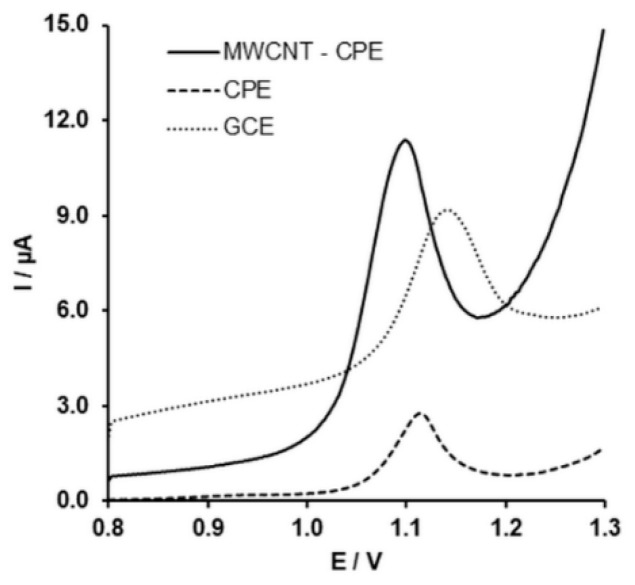
Differential pulse voltammogram (DPV) of 2.5 × 10^−5^ M SIM in 0.1 M H_2_SO_4_ at various electrodes; step potential: 5 mV; modulation amplitude: 100 mV; modulation time: 0.04 s; interval time: 0.1 s.

Figure 7 shows the differential pulse voltammogram (DPV) of SIM on three kinds of electrodes. As seen, the SIM oxidation peak possesses the lowest baseline current in the case of CPE. Compared to CPE and GC, the developed electrode shows a higher current attitude response, which is due to the synergetic effect of the MWCNTs incorporated in the carbon paste. Probably due to the higher hydrophobicity of the carbon paste, the adsorption of the SIM onto its surface takes place, while the presence of the MWCNTs increases the conductivity of the electrode and increases the peak current attitude.

**Figure 8:**
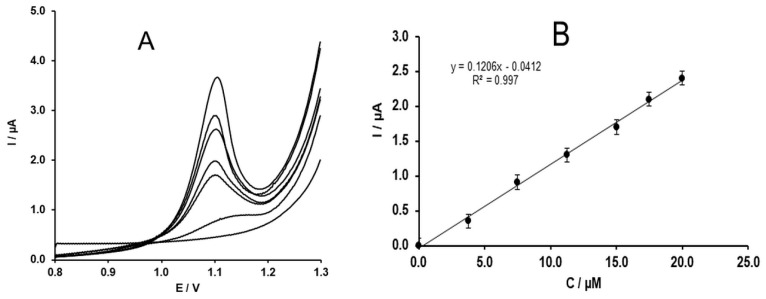
(**A**) DPV of SIM recorded at different concentrations (0, 3.75, 7.5, 11.25, 15, 17.5, and 20 µM); (**B**) concentration dependence of the SIM oxidation peak current.

Moreover, the oxidation peak shifts to the less positive potential at the developed electrode due to the catalytic effect of the MWCNTs. Thus, the developed electrode was used to study the concentration dependence of the peak current. As seen in Figure 8A, the peak current increases linearly with the concentration of the SIM in solution. The concentration dependence of the peak current (Figure 8B) is defined by Equation (5) (R=0.998).

(5) I(µA)=0.1206·C (µM)−0.0531.

### 2.6. Determination of SIM in Pharmaceutical Dosage Form and Its Assay in Spiked Plasma 

**Figure 9:**
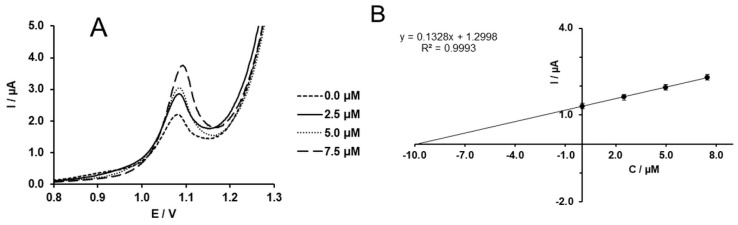
(**A**) DPVs of solution of 7.5 µM SIM prepared in tablet form and three standard additions of 5 µM, 7.5 µM, and 10 µM; (**B**) standard addition curve in which the extrapolations show the initial concentration of SIM in the measuring solution.

The possibility of the application of the developed method for the direct determination of SIM content in a commercial tablet was investigated using the standard addition method without any filtration or other pre-treatments. The results are shown in Figure 9 and Table 1. Based on the obtained results, the determination of SIM in drug dosage form can be carried out with the developed method with good accuracy (relative recovery rate = 99.7%) and reproducibility (relative standard deviation (RSD%) = 1.8). The determination of SIM in human plasma, which is a more complicated matrix, was also performed. The plasma was spiked with a known concentration of SIM and then the spiking recovery rate was determined using the standard addition method. The results are presented in Table 1. The satisfactory nature of the SIM analysis in plasma can be confirmed from the high reproducibility (RSD% = 2.3) and high accuracy of the measurement (spiking recovery rate = 99.5%) (Table 1). It must be mentioned that the concentration of the SIM in plasma is very low (1–15 nM). However, the developed method can be used for the determination of SIM concentration in plasma at levels attributed to the pleiotropic effects (1–50 µM) [8,9,10,11].

### 2.7. Interference Study

The oxidation peak of 5 × 10^−6^ M SIM was recorded in the presence of potential interferents such as ascorbic acid and citric acid in 100-fold higher concentration. It was found that the peak current and potential were not affected due to the presence of these chemicals (Figure S3).

## 3. Materials and Methods

### 3.1. Chemicals

Simvastatin was kindly provided by Prof. Sibel Ozcan (Faculty of Pharmacy, Ankara University, Department of Analytical Chemistry), and its pharmaceutical dosage form (Simgal) was provided by TEVA Czech. Ind. (Opava, Czech Republic). All chemicals for the preparation of buffers and supporting electrolytes were reagent-grade (Sigma-Aldrich, St. Louis, MO, USA). A stock solution of SIM (10^−2^ M) was prepared in ethanol, and the required concentration of SIM was prepared by dilution. The final percentage of ethanol was kept at 10% in all solutions. Solid MWCNTs (purified to more than 95% carbon with an average diameter of 10 nm and an average length of 1.5 µm) were purchased from Sigma. Expanded graphite for preparation of the CPE was purchased from Graphite Tyn Ltd. (Tyn nad Vltavou, Czech Republic). The paraffin oil was also purchased from Sigma. Ferrocene methanol (FcOH), which was used as the mediator for the SECM imaging, was purchased from ABCR GmbH chemicals (Karlsruhe, Germany).

### 3.2. Apparatus

Voltammetric measurements were carried out using a potentiostate/galvanostate AUTOLAB PGSTAT 302 controlled by Nova software (EcoChemie, Utrecht, the Netherlands). The conventional three-electrode configuration for the electrochemical experiments was used. The constructed CPEs and MWCNT-CPEs were used as the working electrodes, while an Ag/AgCl (3 M KCl) electrode and a platinum wire served as the reference and auxiliary electrodes, respectively. The measurements were carried out in one-compartment voltammetric cells (10–20 mL) at conditioned room temperature (23 ± 1 C). The pH measurements were performed using a pH meter Model Sentix 81 (WTW, Weilheim, Germany) with a combined electrode (glass electrode = Ag/AgCl (3 M KCl) reference electrode) with an accuracy of pH ±0.05.

A CHI 900 set-up (CH Instrument Inc., Bee Cave, TX, USA) was used for the scanning electrochemical microscopy (SECM) measurements. A Pt ultra-microelectrode (d = 10 µm) served as the SECM tip (RG factor = 10), while MWCNT-CPEs prepared with different MWCNT mass ratios were used as SECM substrates. The counter electrode was a platinum wire, and the reference electrode was an Ag/AgCl (3 M KCl) electrode. FcOH 1 mM in 0.1 M KCl was used as a redox mediator. All potentials are referred to the reference electrode. The scanning electron microscope MIRA2 LMU (Tescan, a.s., Brno, Czech Republic) was also used for imaging the prepared MWCN-CPE electrodes. An accelerating voltage of 15 kV was applied to produce an electron beam of about 1 nA to visualize the electrode surface at a working distance of 4 mm. Atomic force microscopy (AFM) for the electrode surface characterization was carried out with a dimension FastScan Bio from Bruker (Billerica, MA, USA) operating with Gwydion 2.52 for data visualization [36].

### 3.3. Preparation of the Working Electrode

The MWCNT-modified carbon paste electrodes (MWCNT-CPEs) were prepared by intimately hand-mixing graphite powder and MWCNTs with highly viscous paraffin oil in three different ratios (a: 55%/15%/30%, b: 45%/25%/30%, and c: 35%/35%/30% graphite powder, MWCNTs, and paraffin oil, respectively). All the components were homogenized to obtain a mixture that was subsequently packed into a piston-driven carbon paste holder (with an internal diameter of 2 mm). Whenever needed, the surface of the MWCNT-CPEs was mechanically renewed by extruding ca. 0.5 mm of the paste out of the electrode holder and smoothing with a wet filter paper. To find the optimum combination, various mass percentages of MWCNTs in the paste were utilized and examined.

### 3.4. Pharmaceutical Dosage Form Assay Procedure

Ten tablets of Simgal (each tablet contains 10 mg of SIM) were weighed and finely powdered. An amount of pharmaceutical powder equivalent to 10^−2^ M SIM (10 mL) was dissolved in ethanol. The complete dissolution was ensured by 10 min of sonication. The desired concentration of SIM solution was then prepared by taking the aliquots of the clear supernatant liquor and diluting with the supporting electrolyte. For pharmaceutical dosage form analysis, the standard addition method was carried out, in which a known concentration of SIM solution prepared using the tablet was subjected to three additions of small volumes of stock solution of SIM.

### 3.5. Recovery Studies in Spiked Human Plasma Samples

The human plasma samples, which were obtained from healthy individuals, were stored frozen until the assay. To prepare 2 mL of 10^−3^ M SIM in plasma, after gentle melting at room temperature, the aliquots of plasma were spiked with the required amount of SIM stock solution and treated with 700 µL of acetonitrile as a plasma protein precipitating agent; then, the same plasma sample was added to reach 2 mL. After that, the sample was subjected to 5 min of agitation and then 10 min of centrifugation at 16,500 RCF in order to precipitate the protein residues. The desired concentrations of the plasma samples were prepared by taking the supernatants and mixing them with the required amount of the supporting electrolyte. It must be mentioned that the ethanol percentages in all the analyzed samples were the same.

### 3.6. Procedure

DPV was used for electroanalytical determination of SIM with the following parameters: step potential: 5 mV; modulation amplitude: 100 mV; modulation time: 0.04 s; interval time: 0.1 s. 

To record the approach curve, a potential of 600 mV was applied to the tip where the oxidation of FcOH to FcOH_ox_ takes place. At the conductive surface, the generated FcOH_ox_ is reduced to the parent FcOH, which is transferred to the tip and increases the tip current. On the contrary, where the substrate surface insulates the process, the diffusion of the FcOH from the bulk solution to the tip is limited, which decreases the tip current. 

The probe was then approached to the substrate at a tip scan rate of 0.5 µm∙s^−1^. When the tip is far from the substrate and the potential is applied, FcOH_ox_ is generated and the steady-state current $i_{T,\infty }$ is obtained, which is controlled by the rate of mass transfer by diffusion of FcOH from the bulk solution to the electrode surface.

The current $i_{T}$ increases upon approaching the tip to the substrate because of positive feedback. The approach was interrupted when $i_{T}$ = 1.25 $i_{T,\infty }$. At this magnitude of current, a distance of 10 µm between the tip and the substrate is expected based on the theoretical curve of distance (d) dependency of $i_{T}$ for the utilized ultra-microelectrode (UME) [37]. The constant height mode was used to record the images at a tip scan rate of 1 µm·s^−1^ and an area of 100 μm × 100 μm of the samples.

## 4. Conclusions

The bulk-modified MWCNT–CPEs were prepared and characterized to find the optimum amount of MWCNTs in the paste. The characterization was performed using SECM, SEM, and AFM. Based on the obtained results, the 25% MWCNT–CPE was the optimum electrode in terms of the homogeneity of the prepared paste. Then, the 25% MWCNT–CPE was used for the analysis of SIM in pharmaceutical dosage form, as well as in spiked human plasma. The results confirmed the satisfactory nature of the developed method considering the calculated parameters including bias, relative standard deviation (RSD%), and the recovery rate, as reported in Table 1. The low limit of detection and limit of quantification, in addition to the slope of the calibration curve, proved the sensitivity of the prepared electrode. The advantages of the developed method compared to other electroanalytical methods include simple preparation of the electrode and renewal of the electrode surface, whereas any toxic compounds such as mercury or other heavy metals were not used, and its sensitivity is higher compared to the bare GC and CPE. The developed method offers a simple screening approach for preliminary monitoring. Furthermore, the developed method can be used for rapid but accurate determination of SIM in pharmaceutical dosage form and in plasma samples when its concentration corresponds to the pleiotropic effects. 

## Figures and Tables

**Table 1:** The analytical parameters obtained for the calibration of standards, drug dosage form and plasma sample.

Calibration with Standards		Drug Dosage Form		Spiked Plasma	
Peak potential (V)	1.10	Potential	1.10	Potential	1.10
Linearity range (µM)	3.75–20	Slope	0.1183	Slope	0.154
Slope (µA∙µM^−1^)	0.121 ± 0.005	Intercept	0.8854	Intercept	1.532
Intercept	0.041	Correlation coefficient *r*	0.996	Correlation coefficient *r*	0.999
Correlation coefficient (*r*)	0.998 ± 0.004	Labelled claim (mg)	10	Spiked (µM)	10
LOD ^1^ (µM)	2.4 × 10^−7^	Found (mg)	9.97	Found (µM)	9.95
LOQ (µM)	8.0 × 10^−7^	Recovery rate %	99.7	Bias %	0.5
RSD% (*n* = 8)	4.8	Bias %	0.3	Spiking Recovery rate%	99.5
Confidence interval ^2^	±1.0 × 10^−8^	RSD% (*n* = 3)	1.8	RSD% (*n* = 3)	2.3

^1^ Obtained with 3σ/m (σ: standard deviation, m: slope of the calibration curve); ^2^ confidence interval of 95% = $\frac{t\;s}{\sqrt{n}}$ (*s*: standard deviation, *t*: critical value for eight repetitions: 2.31, *n* = 8 repetitions). RSD%—relative standard deviation.

## Supplementary Materials

The following are available online: Figures S1–S3.
