# Supplementary material for: Preparation and Characterization of Carbon Paste Electrode Bulk-Modified with Multiwalled Carbon Nanotubes and Its Application in a Sensitive Assay of Antihyperlipidemic Simvastatin in Biological Samples
Source: Molecules. 2019 Jun 13;24(12):2215. doi: 10.3390/molecules24122215 (PMC6630724; doi:10.3390/molecules24122215)
Supplement: Supplementary file 1 [file molecules-24-02215-s001.pdf]

Preparation and characterization of carbon paste electrode bulk modified with multiwalled carbon nanotubes and its application in sensitive assay of antihyperlipidemic simvastatin in biological samples

Amir M. Ashrafi, Lukáš Richtera *

Faculty of Electrical Engineering and Communication, Department of Microelectronics, Brno University of Technology, Technická 3058/10,CZ-616 00 Brno, Czech Republic;
amirmansoor.ashrafi@mendelu.cz (A.M.A.); richtera@mendelu.cz (L.R.)
* Correspondence: richtera@mendelu.cz; Tel.: +420-545-133-311

Received: date; Accepted: date; Published: date

Supplementary Materials:

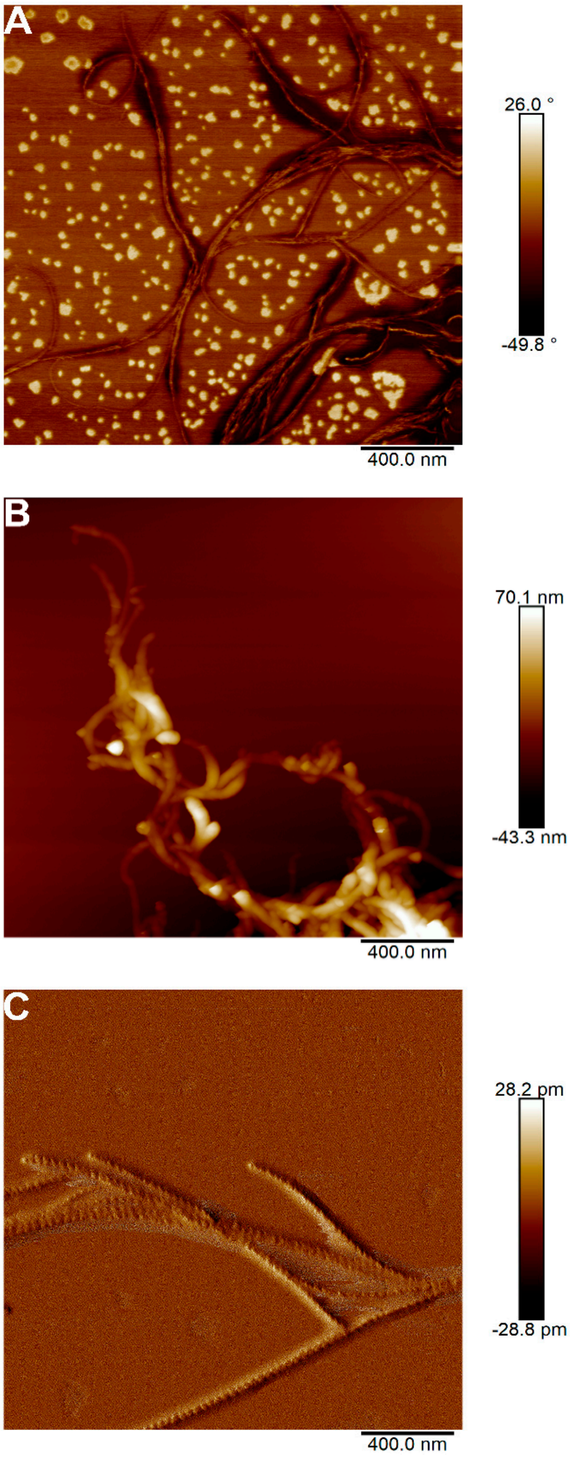

Figure 1. The obtained 2D AFM images of the prepared MW–CPE, A: MW-CPE 15%, B: MW-CPE 25%, and C: MW-CPE 35 %.

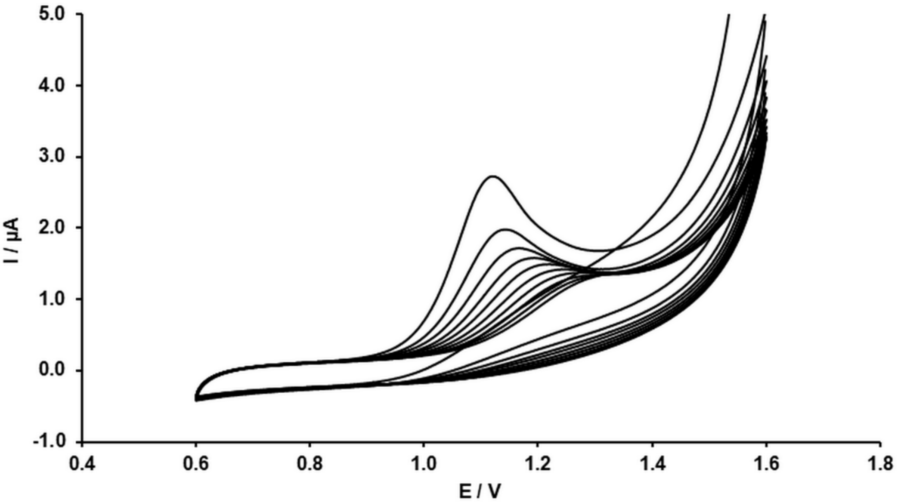

Figure 2. Ten repetitive CVs of 5×10^{-4} M of SIM in 0.1 M H_2SO_4, scan rate 50 mV s^{-1}.

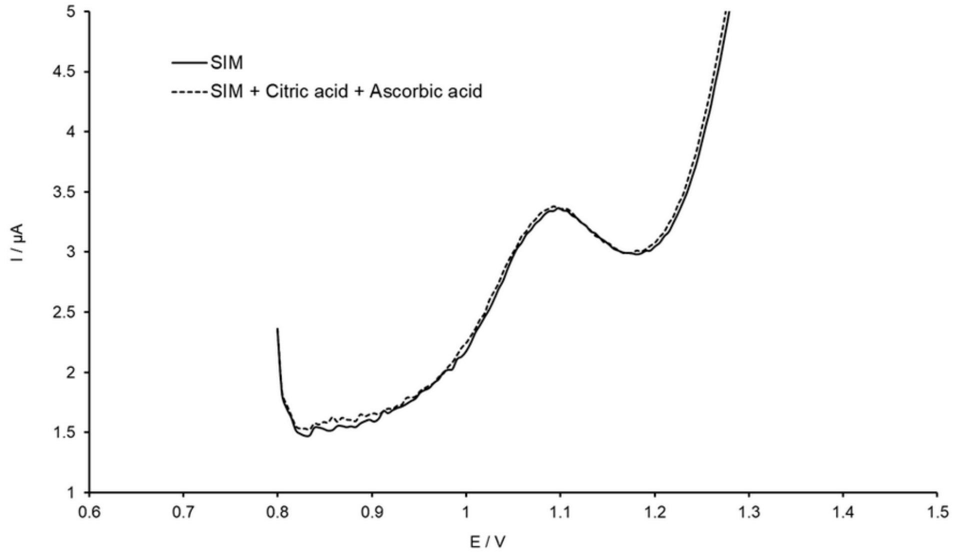

Figure 3. DPVs of solution of 5 µM **of** SIM in without and in presence of 5 × 10^{-4} M of ascorbic acid and citric acid.
